# The impact of PD-L1 on survival and value of the immune check point inhibitors in non-small-cell lung cancer; proposal, policies and perspective

**DOI:** 10.1186/s40425-018-0320-3

**Published:** 2018-02-20

**Authors:** Helmy M. Guirgis

**Affiliations:** Hematology-Oncology Section, Department of Medicine, University of California, Irvine, California USA

## Abstract

**Background:**

The impact of programmed death receptor-ligand1 (PD-L1) on costs and value of the immune check point inhibitors (ICPI) has received minimal attention. Objectives: 1- Design a sliding scale to grade survival in 2nd-line non-small-cell lung cancer (NSCLC). 2- Compare costs and value of Nivolumab (Nivo), Atezolizumab (Atezo) and Pembrolizumab (Pembro) vs. Docetaxel (Doc).

**Methods:**

Previously reported median overall survival (OS) and prices posted by parent company were utilized. The OS gains over controls in days were graded (gr) from A+ to D. Docetaxel costs were calculated for 6–12 cycles and the ICPI for 1 year. Adverse events treatment costs (AEsTC) were reported separately. The cost/life-year gain (C/LYG) was computed as drug yearly-cost/OS gain over control in days × 360 days. The relative value of the ICPI were expressed as $100,000/C/LYG.

**Results:**

Costs of Doc 6 cycles were $23,868, OS/gr 87/C, AEs gr ¾ > 20%, AEsTC $1978 and 6- 12 cycle C/LYG $98,764 -$197,528. Nivo, Atezo and Pembro gr ¾ were < 20% at average costs of $1480. In non-squamous NSCLC, Nivo demonstrated OS/g 84/C and C/LYG $558,326 as compared with 264/A and $177,645 in PD-L1 > 10%. Atezolizumab OS/g were 87/B and C/LYG $551,407 improving in enriched PD-L1 to 162/A and $332,020 respectively. Pembrolizumab in PD-L1 > 1.0% demonstrated OS/g 57/C and C/LYG $659,059 improving in > 50% PD-L1 to 201/A and $186,897. PD-L1 enrichment increased RV of Nivo from 0.18 to 0.56, Atezo from 0.16 to 0.66 and Pembro from 0.15 to 0.53.

**Conclusions:**

Simplified methodology to grade OS and weigh value of anticancer drugs was proposed. In 2nd-line non-squamous NSCLC, value of Doc, Nivo, Atezo and Pembro regardless of PDL-1 expression were limited and modest. Enrichment of PD-L1 resulted in unprecedented OS, improved grades and enhanced value at seemingly justifiable costs.

## Background

Docetaxel (Doc) has been widely used since 2000 in the 2nd-line treatment of patients with metastatic non-small-cell-lung cancer (NSCLC). The median overall survival gain (OS) over best supportive care was 87 days [1]. In 2006, Bevacizumab, a monoclonal antibody against the vascular endothelial growth factor demonstrated a median OS gain of 60 days in 1st-line non-squamous NSCLC [2]. In a landmark study in 2009, the tyrosine kinase inhibitor gefitinib significantly improved the progression-free-survival in epidermal growth factor receptor (EGFR) mutations [3]. The introduction of the immune check point inhibitors (ICPI) changed the landscape of unmutated EGFR- NSCLC treatment. Nivolumab (Nivo) [4, 5] and Pembrolizumab (Pembro) [6, 7] both directed against the program death potein1 (PD-1) and Atezolizumab (Atezo) [8–10] targeting the ligand PD-Ligand 1 (PD-L1) were approved by the Federal Drug Administration (FDA) in 2nd-line. These inhibitors block the PD1 pathway, up-regulate the T cell immunity and allow the immune system to attack tumor cells. The efficacy and safety of the entire ICPI class have been well documented [4–13]. Their cost-effectiveness however has received lesser attention. In the United States (US), an average cost-effectiveness ratio (ACER) of $100,000 has been generally accepted. Simplified methodology to weigh Nivo costs and value was recently described. The yearly-cost/life-year gain(C/LYG) were expressed relative to $100,000 [14, 15]. There is a compelling need for a simplified strategy to facilitate communication of drug outcome and value between medical professionals and patients. Our objectives were: 1-Grade survival gain over control in days 2-Weigh costs vs. value of Nivo, Atezo and Pembro in 2nd-line non-small-cell lung cancer (NSCLC). Docetaxel and Ramu were used as comparators.

## Methods

Drug doses, frequency, OS gains over control and hazard ratios (HR) were quoted from previously published clinical studies. Prices and protocols were utilized as posted by the parent companies. Costs of Nivo 3.0 mg/Kg q 2w intravenously (iv), Doc 75 mg/m2 and Ramu 10 mg/Kg iv q 3 weeks were calculated for 70 Kg patients. Atezolizumab 1200 mg and Pembro 2.0, 10 mg/Kg and 200 mg iv were used q 3w. The OS gains in days were graded on a sliding scale as A+ for OS > 240 to D: < 60 days (Table 1). Adverse events-treatment costs (AEsTC) were reported separately. Docetaxel costs were calculated for 6–12 cycles and the ICPI for 1 year. Costs/life-year gain (C/LYG) were calculated as the drug yearly-cost /OS gain over control in days × 360 days. The relative values (RV) of the ICPI were expressed as $100,000/C/LYG.

**Table 1:** The OS grading System

Grade	OS in days
A+	> 240
A	> 180–240
B	> 120–180
C	60–120
D	< 60

## Results

The OS gains in days by the evaluated drugs were graded on a sliding scale from A+ for OS > 240 to D < 60 (Table 1).

Review of safety of Doc [1] and Ramu/Doc combination [16] revealed AEs of > 20%. Both drugs carry black box warnings, a mechanism designed by the Federal Drug Administration (FDA) to communicate drug safety to patients.

### Docetaxel

The estimated costs of Doc 6 cycles were $23,868, OS/gr 87/C at 6-12 cycle C/LYG $98,764 - $197,528. Based on pre-emptive use of colony stimulating agents and early recognition of peripheral neuropathy, the AEsTC of 6 cycles were $1978 increasing to $4840 for 12 cycles. Long-term treatment costs of delayed and chronic neuropathy were not considered.

### The ICPI

In phase III trials of nivolumab vs docetaxel in previously treated NSCLC, all-grade AEs were less frequent with nivolumab than with docetaxel [4, 5]. Nivolumab, Atezo and Pembro were AEs gr ¾ < 20% [4–10]. In early 2016, the estimated median 4-week costs of the ICPI was $10,077 at a yearly cost of $131,001. The 4-week costs of Nivo 240 mg were later posted as $11,914, Atezo 1200 mg $11,493 and Pembro 200 mg $11,509. At 10 mg/Kg, the 4-week costs of Pembro were $40,135 and Nivo $33,063. Based on early recognition and preventive interventions, the yearly AEsTC did not differ from one ICPI to another and were estimated at $1978.

In Table 2, the OS/g of the squamous histology were 96/B and HR 0.59. The C/LYG were $488,524 and RV 0.20. In non-squamous, the OS/g were 84/C and HR of 0.73. The C/LYG was $558,326 and RV 0.18. There was no reported difference between squamous vs. non-squamous histology using Atezo or Pembro.

**Table 2:** Overall Survival and Value of Docetaxel and Ramucirumab in 2nd-line

Nivo in 2nd-line	OS gains in days/Grade (OS/g) & HR	$4-week Costs	year-Costs	$C/LYG
Generic docetaxel (Doc) 75 mg/m2 vs. supportive care [1]	87/C HR not reported *P* = 0.01	$306	6 cycles: $23,868	$98,764 $197,528
			12 cycles: $47,736	
Ramucirumab (Ramu) + Doc vs. Doc, squamous and non-squamous, (REVEL) [16]	42/D HR 0.86 *P* = 0.0235	$9333	$121,329	$1,039,963

year-Costs = 4-week Costs of $306 × 13 weeks = $3978

year-Costs of 6 cycles = $3978 × 6 = $23,868

*C/LYG* yearly-cost/OS gain in days × 360 days

The costs of AEs treatment (AEsTC) were not included

A summary of the impact of PD-L1 on OS/g and value in non-squamous NSCLC was shown in Table 3.

1. In subset analyses, PD-L1 > 10% enrichment markedly improved Nivo OS/g from 84/C to 264/A+ and RV from 0.18 to 0.56 (Table 4).
2. Atezolizumab: In the POPLAR study most patients had co-expression of PD-L1 on tumor cells and tumor infiltrating lymphocytes [8], the OS/g “in all comers” demonstrated OS/g of 87/C and C/LYG $551,407 and RV 0.16. The results improved in PD-L1 > 50% or tumor infiltrating immune cells (IC) to 348/A+ and RV 0.66 (Table 2).
3. Enrichment of PD-L1 improved Pembro 2.0 mg/Kg OS/g from 57/C to 201/A and RV from 0.15 to 0.53.

**Table 3:** Overall Survival and Value of Nivolumab

Nivo in 2nd-line	OS gains /Grade (OS/g) & HR	C/LYG	Relative Values (RV) (100,000/LYG)
Nivolumab (Nivo) vs. Doc, squamous-NSCLC [4]	96/C HR 0.59 *P* = 0.00025	$488,524	0.20
Nivo vs. Doc, non-squamous- NSCLC, CheckMate 057 [5]	84/C HR 0.73 *P* = 0.0016	$558,326	0.18
Subset analysis in > 10% positive PD-L1 ChecMate 057 [5]	264/A + HR 0.27	$177,645	0.56

Nivolumab was tabulated separately since it was the only ICPI which showed OS difference between squamous- and non-squamous histology. Nivo, Atezo and Pembro demonstrated AEs gr ¾ of < 20%, maintained/or improved the QoL and carried no black box warnings. The adverse events treatment costs (AEsTC) represented a small and insignificant fraction of drug costs and were not included in drug costs. The C/LYG were weighed relative to $100,000. Relative values (RV) were computed as $100,000/C/LYG

**Table 4:** Overall Survival and Value of Atezolizumab and Pembrolizumab

Drug and setting	OS gains in days/Grade(OS/g) & HR	C/LYG	RV
^a^Atezolizumab (Atezo) vs. Doc irrespective of PD-L1, vs. Doc (phase II, POPLAR) [8]	87/C HR 0.73 *P* = 0.040	$618,244	0.16
Atezo, low or undetectable PD-L1 vs. Doc, Phase III OAK [9, 10]	111/C HR 0.75	$475,265	0.21
Atezo, PD-L1 > 1.0% tumor cells (TC) or in tumor-infiltrating immune cells (IC) vs. Doc [10]	162/B HR 0.74 *P* = 0.0102	$325,644	0.30
Atezo, PD-L1, TC or IC > 5% vs. Doc, [10]	165/B	$319,974	0.31
Atezo, PD-L1 > 50% or IC > 10% vs. Doc	348/A + HR 0.41	$151,193	0.66
Pembrolizumab (Pembro) PD- L1 > 1.0% positive vs. Doc KEYNOTE − 010 [7] Subset analysis	57/D HR 0.71 *P* = 0.0008	$659,059	0.15
Pembro 10 mg/Kg, PD-L1 1.0% positive, KEYNOTE − 010	126/B HR 0.61	$1,490,729	0.07
Pembro 2.0 mg/Kg, > 50% positive KEYNOTE − 010	201/A HR 0.54	$186,897	0.53

^a^The average OS gains of Atezo in the POPLAR and OAK were 99 days at C/LYG of $551,407 and RV 0.19

## Discussion

The present investigation was prompted by the rising costs of anti-cancer drugs, stagnant or diminishing value and widening gap in communication of cost and value issues between physicians and patients [20, 21]. Progress in drug development was achieved at high costs. The American Society of Clinical Oncology (ASCO) [17] and the European Society of Clinical Oncology (ESMO) [18] emphasized the role of value in the overall economic formulary [19]. Our primary objectives were to design a grading system to measure OS and weigh costs vs. value of the ICPI in NSCLC. With the dropping out of Ramu from 2nd-line, Doc remained the only comparator.

### Methodology

The unbiased and reliable OS endpoint was used throughout the present investigation. There is growing recognition among the oncology community that OS gains by anticancer drugs of < 2 months might not be clinically significant. In our study, grade D was assigned to < 60 days and A+ to > 240 days. Our goal was to facilitate full disclosure and transparency of outcome, costs and value to patients [20, 21]. The proposed methodology was not designed for medical economists but was rather geared towards the community oncologists. The simplicity of the grading system could enable oncologists, pharmacists and nurses to rapidly calculate drug value. The value was expressed relative to $100,000, the average acceptable ACER in the US. Admittedly, there are drawbacks limiting applications of the proposed methodology. Costs of hospitalization, drug administration, long-term treatment of chronic AEs and global QoL measures were not included. Fees for professionals including physicians, pharmacists and nurses were not considered. The methodology also failed to capture and capitalize on the longer duration of response of the ICPI over Doc.

Previously published methodology measuring differences in cost vs. outcome between 2 interventions [22, 23] could not be applied since in the present investigation 5 drugs were compared. Generic Doc costs were, not surprisingly much cheaper as compared with the ICPI. There was a non-significant cost difference between Nivo, Atezo and Pembro. The 4-week costs of Doc were $306, Nivo 240 mg $11,914, Atezo 1200 mg $11,493 and Pembro 200 mg $11,509. The incremental increase in cost per one-day OS gain was therefore used as the basis of our calculations. Costs, value, hazard ratios (HR) and median OS of Nivo at 12 months-milestone have recently been reported in a wide variety of cancer [15].

### AEs

Docetaxel demonstrated AEs gr 3/4 of > 20%. The average AEsTC of 6 cycles were $1978 and 12 cycles $4940. The AEsTC could be burdensome and costly [24]. The cascade of neutropenia to febrile neutropenia, the non-hematological AEs and peripheral neuropathy have been well documented [1].

The gr ¾ AEs of the ICPI were generally < 10% [4–10]. The oral and iv steroids used for treatment of AEs were inexpensive. The costs of treatment represented an insignificant fraction of the total drug costs. With early use of scans and timely interventions, the average yearly AEsTC per patient treated by the ICPI were estimated at $1480. In CheckMate 017 in the squamous-NSCLC, the AEsTC of Nivo were $439 vs. docetaxel of $7024. In CheckMate 057 in non-squamous, Nivo AEsTC were $518, vs. Doc $5940 [25]. There has been wide spread awareness of the potential hazards with continued gain in experience and management of AEs since the introduction of bevacizumab [26], Doc and the ICPI.

In our study, Nivo demonstrated discernible value improvement of the squamous over the non-squamous histology. Atezolizumab and Pembro seemed to be equally effective irrespective of histology. Of interest, there is no documentation yet of benefit by PD-L1 enrichment in the squamous type. The latter is generally considered more homogenous with a higher incidence in non-smokers and males than the non-squamous.

### Cost vs. value

The balance between drug costs and value is sensitive but elusive. The debate seems to continue [27]. Costs need to be maintained at certain levels for drug companies to proposer and keep their innovative edge. It is estimated that 1 billion dollars is spent to successfully bring a formula from the drawing boards to pharmacy shelves. Nonetheless, the price tags of 10 mg/Kg of Nivo and Pembro could dampen their use. The affordability of the ICPI in combination with other immune drugs e.g. Ipilimumab would be negatively impacted and curtailed using relatively high doses and frequent administrations. Such costs could be only tolerated if long-term disease control and/or cure are achieved.

The pendulum however has recently tipped in favor of value since ASCO and ESMO issued their initiatives in 2013 [17, 18]. The National Institute for Health and Care Excellence (NICE) in the United Kingdom stipulates that ACER interventions ought not to exceed 20,000–30,000 pounds per quality-adjusted life-year gained. Of note, Nivo approval by NICE is pending and negotiations are still continuing. It would be helpful to oncology professionals, patients, drug authorities and society if the FDA could give further guidance on value. At present, the US law prohibits discussion of drug costs during the approval process.

### PD-L1 biomarker and drug value

In the early clinical studies of the ICPI in NSCLC various PD-L1 expression in tumor cells and infiltrating immune cells. At present, PD-L1 levels are being standardized and levels expressed as tumor proportion scores (TPS). There is up to 30% response in patients with PD-L1 enhanced expression. The findings of durable responses in non-enriched PDL-1 are indicative of the “imperfections” of PDL-1 marker. The tumor mutation load in combination with PDL-1, are emerging as more precise predictive markers [28].

The present study was the first to demonstrate that PDL-1 enrichment resulted in improved OS and enhanced value in NSCLC. The OS increased from < 100 days with C and D grade to > 150–300 days with B and A+. The OS increase by PDL-1 enrichment was unexpected and unprecedented in an incurable disease as NSCLC. From an economic point of view, it would be prudent to spend upfront few hundred dollars on a reliable predictive marker or set of markers than to bear the costs of treating “all comers”. This strategy would spare the non- responders the high costs and unnecessary toxicity of ineffective therapy.

### Duration of treatment

The ICPI treatment paradigm is evolving. The current recommendations on use of the ICPI are to continue treatment till AEs occurrence, progression or beyond progression. Costs and to a lesser extent AEs would increase with extended use. The duration of treatment comes down to a choice between cost vs. value and is presently an area of active investigation. It remains to be seen whether the incremental survival benefit by prolonged use ICPI could justify the additional yearly- costs of $131,000.

### Policies and perspectives

The FDA has recently adopted a new trend of approval of new drugs based on early endpoints other than survival. The present methodology could be modified to use disease control rates and other endpoints after adjustments with appropriate correction factors. Higher doses and longer duration of treatment by costly drugs need to be scrutinized before utilization.

In 2nd-line non- mutated NSCLC, the superiority of ICPI over chemotherapy has been generally accepted despite concerns on cost issues. Costs of Nivo and Pembro were described by Salts [29] as thousands of times the cost of gold.

In 1st-line, Pembro in combination with pemetrexed and carboplatin without PD-L1 enrichment has recently demonstrated higher response rates with deeper and longer duration of response [30]. The overall survival data have not yet matured. Clearly, PD-L1 is not the whole story. Tumor mutation burden can be used as well to decide on immunotherapy approaches [31]. Enthusiastic use of the ICPI however needs to be tempered by the economic pressures of countries and patients with limited resources. With the upcoming waves of generic chemotherapeutic drugs including pemetrexed, the role of Doc and many others is not yet dead but just diminished [32].

## Conclusions

Simplified methodology to grade OS and weigh value vs. costs of anticancer drugs was proposed for community oncologists. The results suggested that value and costs were essential in securing a fair and equitable economic balance between consumers and drug companies. Doc demonstrated distinct cost and value advantages. Its AEs and impaired QoL were serious impediments. In 2nd-line, value of Nivo, Atezo and Pembro regardless of PDL-1 expression were rather limited and modest. Enrichment of PDL-1 resulted in unprecedented OS, improved grades and enhanced value at seemingly justifiable costs. The consistency of our results could give credence to the conclusions. The non-uniform testing of PDL-1 testing, subset analysis and scanty available comparative data precluded favoring one ICPI over the other.

## Abbreviations

ACER: Average cost-effectiveness ratio
AEs: Adverse events
AEsTC: Adverse events treatment costs
Atezo: Atezolizumab
C/LYG: Cost/Life-year gain
CI: Confidence Interval
EGFR: Epidermal growth factor receptor
FDA: Federal Drug Administration
Gr: Grade
HR: Hazard Ratio
ICPI: Immune check point inhibitors
Nivo: Nivolumab
NSCLC: Non-small-cell lung cancer
OS: Overall survival
PD-1: Program death potein1
PD-L1: Programmed death receptor-ligand1
Pembro: Pembrolizumab
QoL: Quality of Life
Ramu: Ramucirumab
RV: Relative values
TPS: Tumor Proportion Score
